# Codon Optimization Significantly Improves the Expression Level of a Keratinase Gene in *Pichia pastoris*

**DOI:** 10.1371/journal.pone.0058393

**Published:** 2013-03-05

**Authors:** Hong Hu, Jie Gao, Jun He, Bing Yu, Ping Zheng, Zhiqing Huang, Xiangbing Mao, Jie Yu, Guoquan Han, Daiwen Chen

**Affiliations:** Institute of Animal Nutrition, Sichuan Agricultural University, Ya’an, Sichuan, P. R. China; Instituto Butantan, Brazil

## Abstract

The main keratinase (*kerA)* gene from the *Bacillus licheniformis* S90 was optimized by two codon optimization strategies and expressed in *Pichia pastoris* in order to improve the enzyme production compared to the preparations with the native *kerA* gene. The results showed that the corresponding mutations (synonymous codons) according to the codon bias in *Pichia pastoris* were successfully introduced into keratinase gene. The highest keratinase activity produced by *P. pastoris* pPICZαA-kerAwt, pPICZαA-kerAopti1 and pPICZαA-kerAopti2 was 195 U/ml, 324 U/ml and 293 U/ml respectively. In addition, there was no significant difference in biomass concentration, target gene copy numbers and relative mRNA expression levels of every positive strain. The molecular weight of keratinase secreted by recombinant *P. pastori* was approx. 39 kDa. It was optimally active at pH 7.5 and 50°C. The recombinant keratinase could efficiently degrade both α-keratin (keratin azure) and β-keratin (chicken feather meal). These properties make the *P. pastoris* pPICZαA-kerAopti1 a suitable candidate for industrial production of keratinases.

## Introduction

Keratins are major component of cuticulum i.e. feathers, wool, scales, hair, hoofs, nails and so on. Due to intensive cross-linking by hydrogen, hydrophobic and cystine disulfide bonds, keratins are recalcitrant and poorly degraded by most proteinases, e.g. papain, collagenase, pepsin and trypsin [1]–[2]. In spite of the considerably stability, keratin can be efficiently degraded by many microorganisms because of the secretion of keratinases [2]–[3].

Keratinases are metallo or serine proteinases which can degrade the insoluble structure forming keratin substrates. The keratinases obtained from various sources have been widely used in medicine, detergent, cosmetics, leather and biodegradable plastic manufacture [3]–[4]. Keratinases were also used to degrade the prion for treatment of bovine spongiform encephalopathy, scrapie and human Creutzfeldt-Jakob disease [5]. Moreover, keratinases have been developed by poultry and feed industries to improve the properties of poultry diets [6]–[7]. Therefore, the requirement for keratinases has been recently increased. A myriad of bacteria, fungi and actinomycetes were found to naturally secrete keratinases [2]–[4].

*Bacillus licheniformis* has been known as potential host to produce efficient keratinolytic enzymes for industrial applications [8]–[10]. The keratinase (*kerA*) gene of *B. licheniformis* has been sequenced and cloned [11]–[12]. The molecular mass of mature keratinase is about 30–33 kDa [8]–[9]. The keratinase presents an optimum activity around 50–60°C [8]–[9], [13]. The isoelectric point and optimum pH of keratinase are 7.25 and 7.5, respectively [8]–[9]. Due to relatively high enzyme production costs, the industrial applications of keratinase produced by *Bacillus licheniformis* are still not extensive. Moreover, the keratinases secreted by *B. licheniformis* are usually contaminated by other enzymes (e.g. amylase, mannase, cellulolytic enzyme, etc.), which complicate the purification process.

Recently, production of keratinases using heterologous expression systems such as fungi and bacteria have attracted considerable research interests due to high level of protein yield and high purity. One of the most attractive expression systems is the yeast *Pichia pastoris*. It combines many advantages of the eukaryotic protein expression systems including protein posttranslational modification, processing, folding and so on, meanwhile its manipulation is as easy as *E. coli* expression system. Compared to other eukaryotic protein expression systems, the *P. pastoris* expression system is easier, faster, less expensive and higher expression [13]–[14]. Many proteases from bacteria, fungi, actinomycetes and mammal have been successfully expressed in *P. pastoris* system [15]–[16].

It is well known that heterogeneous protein expression level in *P. pastoris* strongly depends on the biased codon usage [17]–[19]. This problem is minimized by codon optimization technique. Previous studies demonstrated that the codon optimization technique greatly increases (about 1- to 10-fold) the foreign proteins expression in *P. pastoris* [20]–[21]. In this study, the *B. licheniformis* keratinase gene was cloned and optimized using different codon optimization strategies. All mutated keratinase genes were expressed in *P. pastoris*. The aim of the present study was to develop a high-level expression system of keratinase. Furthermore, the biochemical properties of the purified recombinant keratinase including substrate specificity and amino acid sequence were examined. To our knowledge, this is also the first report to improve keratinase production by codon optimization strategies in *P. pastoris.*

## Materials and Methods

### Plasmids, Reagents, Media and Strains

*B. licheniformis* S90 which could efficiently degrade the feathers was stored in our laboratory [22] and grown at 37°C for 2–3 d on feather medium [22]. *E. coli* DH5α was grown at 37°C for 12–18 h in Luria-Bertani (LB) broth or low sault LB media. *P. pastoris* X-33 was from our laboratory and cultivated at 30°C for 2–3 d in YPD (1% yeast extract, 2% glucose and 2% peptone) medium or agar. The plasmids pMD20-T was a T-cloning vector and obtained from TaKaRa (China). The pPICZαA was an expression vector for secretion in *P. pastoris* and purchased from Invitrogen (USA). The pMD20T-GAP plasmids was established and stored in our laboratory (unpublished data).

Total genomic DNA was extracted from *B. licheniformis* S90 using TIANamp Bacteria DNA kit (Tiangen, China). Plasmid DNA was extracted from *E. coli* by Plasmid Mini kit I (Omega, USA). The T4 DNA ligase and restriction enzymes were obtained from NEB (USA) and TaKaRa (China) respectively.

### *B. licheniformis* Keratinase Gene (kerAwt) Amplification and Cloning

The DNA fragment encoding the *B. licheniformis* S90 *kerA* pro-enzyme was isolated by PCR with the primers kerAwtF (5′-GCTGGTACCGCTCAACCGGCGAAAAATGT-3′, *Kpn*I restriction site underlined) and kerAwtR (5′-CGAGCGGCCGCTTGAGCAGCAGCTTCGACATTGAT-3′, *Not*I restriction site underlined). Both primers were designed according to the nucleotide sequence of the *B. licheniformis* S90 keratinase gene, as previous work in our laboratory (GenBank accession no. JN859581). The PCR reaction mixtures (50 µl) contained approx.15 ng of *B. licheniformis* S90 DNA, 0.2 µM each primer and 25 µl PCR premix (TaKaRa, China). The thermal program included 1 cycle of 8 min at 95°C, 32 cycles of (45 s at 94°C, 45 s at at 59°C, and 1.5 min at 72°C),and 1 cycle of 12 min at 72°C. The purified DNA product was inserted into plasmid pMD20-T and sequenced by TaKaRa (China).

### Design of the Mutant *kerA* using Different Codon Optimization Strategies

The main keratinase gene of *B. licheniformis* S90 was optimized by partly replacing the *P. pastoris* preferred codons (synonymous codons). In this study, there were two codon optimization strategies–*kerAopti1* and *kerAopti2* (Table 1). The corresponding mutations were introduced into k*erA* by site-directed mutagenesis with the MutanBEST kit (TaKaRa, China) following the manufacturer’s protocol. Both *kerAopti1* and *kerAopti1* genes were inserted into plasmid pMD20-T.

**Table 1:** The primers and site-directed mutable points of mutant keratinase genes.

Mutant gene	Primer	Sequences of primers^a^	Mutable points
*kerAopti1*	kerAopti1F	5′-CCAGCTAAAAATGTTGAAAGGGA-3′	CCG/CCA (Pro-3); GCG/GCT (Ala-4)
	kerAopti1R	5′-GAGCGGTACCAGCAATCC-3′	
*kerAopti2*	kerAopti2F	5′-GTCAGAAACAGACTCTCCAGCAC-3′	CCG/CCA (Pro-3); GCG/GCT (Ala-4); CGC/AGA (Arg-322); CGT/AGA (Arg-324)
	kerAopti1R	5′-TTGTGAAGCTGAAAGGTTCGGATG-3′	

a The mutable points are underlined.

### Construction of the Recombinant Plasmids

A high effective shuttle vector, pPICZαA, was used to express keratinase gene in *P. pastoris*. The plasmids pMD20T-kerAwt, pMD20T-kerAopti1 and pMD20T-kerAopti2 were digested with *Not*I and *Kpn*I, and the isolated DNA fragments were recovered and inserted into pPICZαA vector. The recombinant plasmids, pPICZαA–kerAwt, pPICZαA–kerAopti1 and pPICZαA–kerAopti2, were transformed into *E. coli* DH5α on low salt LB agar plates which contained 25 µg/ml zeocin. The positive clones were identified by colony PCR and sequencing.

About 10–15 µg pure vector pPICZαA containing *kerAwt*, *kerAopti1* and *kerAopti2* were linearized using *Sac*I prior to integration into *P. pastoris* by electroporation (1800 kV, 50 µF, 186 Ω. Bio-Rad, USA). The positive clones were chosen on the YPD-zeocin plates (100 µg/ml zeocin) at 30°C and identified by colony PCR and sequencing.

### Expression of Recombinant Keratinase in *P. pastoris*

A single positive colony was cultivated in 25 mL of BMGY (1% glycerol, 2% peptone, 1% yeast extract, 4×10^−5%^ biotin, 1.34% YNB and pH 6.0, 100 mM potassium phosphate) medium at 29°C under constant agitation at 200–220 rpm until an OD600 = 3–5 was reached (approx. 18–20 h). Then, the cells were collected by centrifugation and resuspended in 50 mL BMMY (1.5% methanol, 2% peptone, 1% yeast extract, 4×10^−5%^ biotin, 1.34% YNB and pH 6.0, 100 mM potassium phosphate) medium. Cultures were induced with absolute methanol to a final concentration of 1.5% every day. The expression culture supernate was harvested every 24 h and stored at −70°C before analysis.

### Protein Purification

Recombinant *P. pastoris* strains were cultivated under the optimized conditions. The expression culture supernatant was clarified by centrifugation and concentrated approx.10-fold via ultrafiltration (MW cut-off, 10000 Da, Millipore, Germany). The supernate containing recombinant keratinase was purified by 2 ml Ni^2+^-chelating chromatography according to the manuals (Bio-rad, USA). The elution buffer (pH 8.0, 50 mM sodium phosphate, 500 mM imidazole and 300 mM NaCl) containing purified keratinase was stored at −70°C before analysis.

### Glycoprotein Staining and Deglycosylation of Keratinase

The SDS-PAGE analysis of purified keratinase with glycoprotein staining experiment was performed using glycoprotein-stain kit (Sangon Biotech, China). The purified enzyme was deglycosylated by Endoglycosidase H_f_ at 37°C for 3 h following the manufacturer’s instructions (NEB, USA). The deglycosylated proteinase was determined by SDS-PAGE.

### SDS–PAGE

SDS–PAGE was carried out on a 12% running gel and stained with Coomassie Blue [23].

### Analysis of *kerAwt*, *kerAopti1* and *kerAopti2* Gene Copy Numbers

*kerAwt*, *kerAopti1* and *kerAopti2* gene copy numbers of generated recombinant *P. pastoris* strains were detected by the previous reports [24]–[26]. Genomic DNA was extracted from positive *P. pastoris* transformants with the yeastgen DNA kit (Cwbiotech, China) according to the instruction manuals. The modified real-time quantitative PCR (qPCR) method is used to determine copy numbers in this study. The glyceraldehyde-3-phosphate dehydrogenase (*GAP*) gene of *P. pastoris* was used as reference gene. All primers were deseigned by Primer 5.0 software (Table 2). Real-time qPCR was performed in a total volume of 10 µl which contained 1µl of DNA, 0.4 µl of 10 µM each primer, 3.2 µl of ddH_2_O and 5µl of 2×SYBR® Premix Ex Taq™ II (TaKaRa, China). All real-time PCR reactions were carried out in triplicate on CFX96™ Real-Time PCR Detection System (Bio-rad, USA) using the following program: 95°C for 30 s, 40 cycles of 95°C for 10 s and 60°C for 30 s. Specificity of amplicons was examined by melting curve analysis after 40 cycles and by agarose gel electrophoresis analysis.

**Table 2:** Primer sequences for quantitative real-time PCR.

Gene	Primer	Sequence 5′-3′	Product size (bp)
*kerA*	RT-kerAF	GTAAAAGTAGCCGTCCTG	174
	RT-kerAR	CAACGCCTAATACACCC	
*GAP*	RT-GAPF	ATGACCGCCACTCAAAAG	98
	RT-GAPR	CACCAGTGGAAGATGGAAT	
*ACT1*	RT-ACT1F	ACACACAGTGTTCCCATCGGT	231
	RT-ACT1R	AAGAACTGGGTGCTC TTCTG	

The pMD20T-kerA and pMD20T-GAP plasmids were used to construct the standard curve. Brieﬂy, the double standard curves of *kerA* and *GAP* were established using 10-fold serial dilutions of the above plasmids ranging from 10^3^ to 10^8^ copies/µl. The cycle threshold (C_t_) values of real-time qPCR in every dilution were determined thrice and plotted against the logarithm of the corresponding template gene copy numbers. Each standard curve was generated by linear regression of the plotted points. The total gene copy numbers of *GAP* and target gene in the genomic DNA sample were determined by relating the C_t_ values to the standard curves. Finally, the target gene copy numbers integrated in the genome of recombinant *P. pastoris* would be calculated by the target gene/GAP copy numbers ratio because *P. pastoris* contains only one copy *GAP* [27].

### Analysis of *kerAwt*, *kerAopti1* and *kerAopti2* mRNA Expression Levels

The real-time qPCR method is used to determine *kerAwt*, *kerAopti1* and *kerAopti2* mRNA expression levels in present study. Total RNA was extracted from positive recombinant *P. pastoris* induced with 1.5% methanol for 96 h using RNAiso plus (TaKaRa, China). The reverse-transcription of total RNA (1 µg) was performed by PrimeScript® RT reagent kit with gDNA Eraser (Perfect Real Time) (TaKaRa, China). All primers were deseigned by Primer 5.0 software (Table 2). The reaction mixtures and program of real-time qPCR were identical to the above method. For normalization, the *ACT1* gene (housekeeping gene) was used as the endogenous control gene. The relative gene mRNA expression (GE) levels for target gene of different strains were treated using the methods from Pfaffl [28]. In this case, the transcription levels of target genes were determined.

### Keratinase Activity Assay

After removal of cells by centrifugation, keratinase activity was detected by the previous method [9]. Brieﬂy, 0.2 ml protease was mixed with 20 mg of substrate keratin azure (Sigma-Aldrich, USA) and 3.8 ml of 0.05 M Tris-HCl buffer (pH 7.5) in a shaking incubator (220 rpm) at 50°C for 1 h. One unit of keratinase activity was defined as the quantity of protease that caused an increase in 0.01 absorbance units (595 nm, A_595_) for 1 h.

In addition, the activity of keratinase was also detected using chicken feather meal as substrate [9]. The enzyme (0.2 ml) was mixed with 20 mg of feather meal and 3.7 ml of 0.05 M Tris-HCl buffer (pH 7.5) in a shaking incubator (220 rpm) at 50°C for 1 h. Then, 0.1 ml 100% (w/v) trichloroacetic acid was added to the above mixture to terminate the reaction. After incubation for 1 h at 4°C, the reaction supernate was collected by centrifugation. One unit of keratinase activity was defined as the quantity of protease that caused an increase in 0.01 absorbance units (280 nm, A_280_) for 1 h.

### Determination of Optimal Temperature and pH

The keratinolytic optimum temperature was examined at 30 to 80°C and pH 7.5. The optimal pH of its activity was measured at the detected optimum temperature by using buffers with pH ranging from 5.0 to 10.0. The used buffers were 0.05 M citrate phosphate buffer (pH 5–6) and 0.05 M Tris-HCl buffer (pH 7–10).

The temperature stability of keratinase was detected by heating protease at various temperatures (50, 60 and 70°C) for 30 min, and the residual activity was examined at 50°C and pH 7.5 for 1 h. To determine the keratinolytic thermal stability, the purified protease sample was incubated 0.05 M Tris-HCl buffer (pH 7.5) in the absence of keratin azure at 50°C for 30, 40, 50, 60, 90 and 120 min before detecting its activity. All assays were carried out in quadruplicate.

## Results and Discussion

### Cloning of kerAwt, kerAopti1 and kerAopti2 Gene

To facilitate the protein purification, the pro-enzyme sequences of *kerAwt*, *kerAopti1* and *kerAopti2* were cloned in fusion with the yeast alpha-factor signal sequence, which allows secretion of pro-enzyme in to the culture medium. The transcription of the gene fusion was under the control of alcohol oxidase 1 promoter (AOX1). As expected, three kinds of keratinase genes (1050 bp) were cloned into pMD20-T simple vector. The nucleotide sequences of keratinase genes indicated that the corresponding mutations were successfully introduced into *kerA*. However, all keratinase genes encoded the same mature protein (350 amino acids) and had the triad of catalytic residues including Asp-32, His-63 and Ser-220 [11].

### Real-time qPCR Approach for Copy Number and Gene Expression Determination

Previous studies suggested that the copy numbers and transcription levels of heterologous gene would influence its expression level in *P. pastoris* [24]–[26]. Therefore, the keratinase gene copy numbers and transcription levels of every positive strain were also determined in this work.

**Figure 1:**
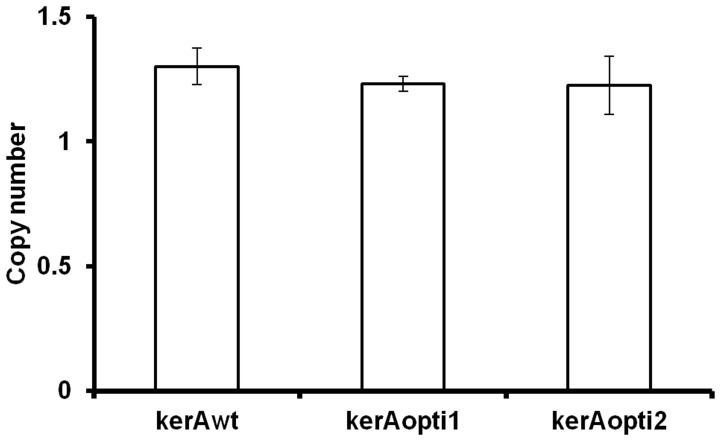
Comparative the target gene copy numbers of *P. pastori* pPICZα-kerAwt, pPICZα-kerAopti1 and pPICZα-kerAopti2. The values represent means ± SD (n = 3). Results showed no significant difference in gene copy numbers of every positive transformant (P>0.05).

Compared to dot/slot blotting and southern blotting used for heterologous gene copy numbers assay in *P. pastoris* system, the real-time qPCR was easier, faster and most importantly, absolute quantitative methods [24], [26]. In this work, a modified real-time qPCR method was used to detect the *kerAwt*, *kerAopti1* and *kerAopti2* copy numbers in transformants [24]–[26]. The double stand curves for *kerA* and *GAP* were constructed by 10-fold serial dilutions of pMD20T-kerA and pMD20T-GAP plasmids. A high correlation between C_t_ values and the copy numbers was presented for both *kerA* and *GAP* determination (R^2^>0.999). The target gene copy numbers in the genome of selected transformants were then detected with the target gene/GAP copy numbers ratio because *P. pastoris* contains only one copy *GAP* within its genomic DNA [27]. Figure 1 showed that there was no significant difference (P>0.05) in the target gene (*kerAwt*, *kerAopti1* and *kerAopti2*) copy numbers of recombinant *P. pastoris* strains.

**Figure 2:**
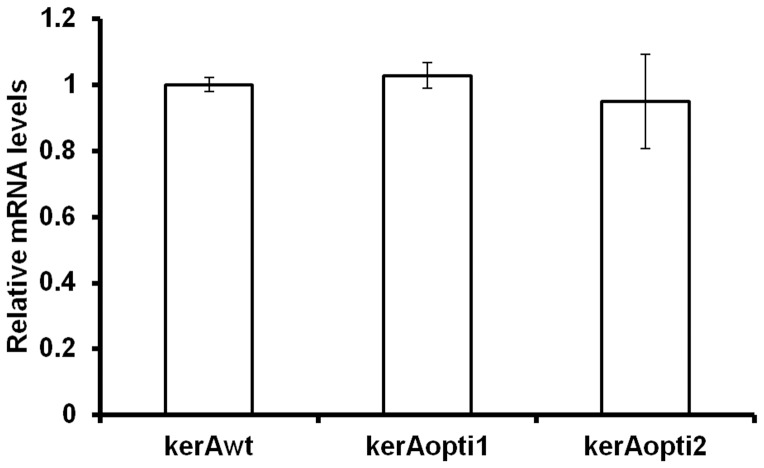
Comparative the target gene relative mRNA levels of *P. pastori* pPICZα-kerAwt, pPICZα-kerAopti1 and pPICZα-kerAopti2. The values represent means ± SD (n = 3). Results showed no significant difference in target gene relative mRNA levels of every positive transformant (P>0.05).

In the present work, the transcription levels of target genes were also determined. The mRNA expression levels of *kerAwt*, *kerAopti1* and *kerAopti2* were detected by real-time qPCR according the method from Pfaffl [28]. Results showed that no significant difference (P>0.05) was observed in the target gene (*kerAwt*, *kerAopti1* and *kerAopti2*) mRNA expression levels of each transformants (Figure 2).

### SDS-PAGE Analysis of the Keratinase from Recombinant *P. pastoris*

**Figure 3:**
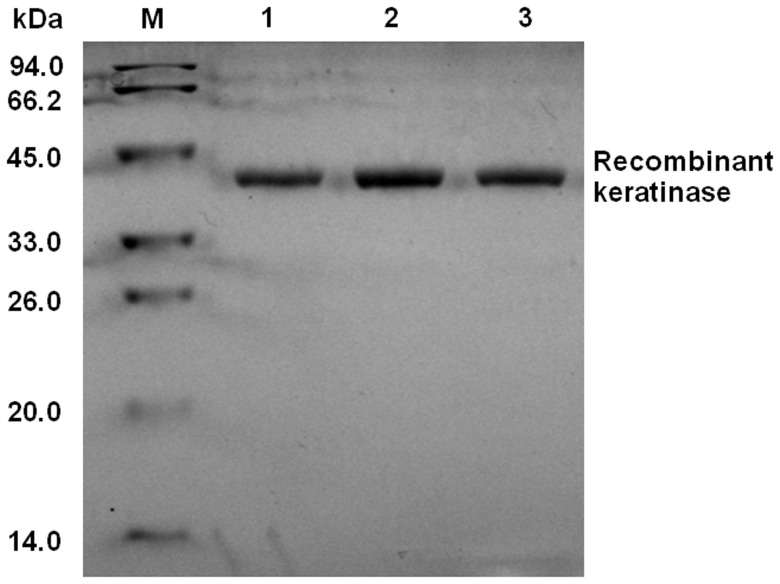
SDS-PAGE analysis. Lane M: protein MW markers (14–94 kDa); Lane 1: the purified keratinase of *P. pastoris* pPICZα-kerAwt; Lane 2: the purified keratinase of *P. pastoris* pPICZα-kerAopti1; Lane 3: the purified keratinase of *P. pastoris* pPICZα-kerAopti2.

The recombinant *P. pastoris* pPICZαA-kerAwt, pPICZαA-kerAopti1 and pPICZαA- kerAopti2 were cultivated under the optimized conditions. The expression supernatant containing recombinant keratinase was successfully purified by Ni^2+^-NTA affinity chromatography, since only one band with MW approx. 39 kDa was presented on SDS-PAGE gel (Figure 3 lanes 1–3). However, the keratinase secreted by *B. licheniformi*s has a MW of 33 kDa [8]. This was probably caused by N-glycosylation [16]. The keratinase amino acid sequence from *B. licheniformis* S90 has 6 potential glycosylation sites (N-glycosylation sites: 152-N, 293-N and 315-N; O- glycosylation sites: 79-T, 231-S and 327-S) and *P. pastoris* is capable of adding N- and O- linked oligosaccharide chains to expressed proteins [29]–[30]. Therefore, the molecular mass of recombinant keratinase from *P. pastoris* was greater than that secreted from *B. licheniformis*. Similar results have been derived for the difference between the MW of the keratinase of *B. licheniformis* PWD-1 and *B. licheniformis* MKU3 expressed in *P. pastoris* and the calculated weight from the keratinase coded protein [9], [13].

**Figure 4:**
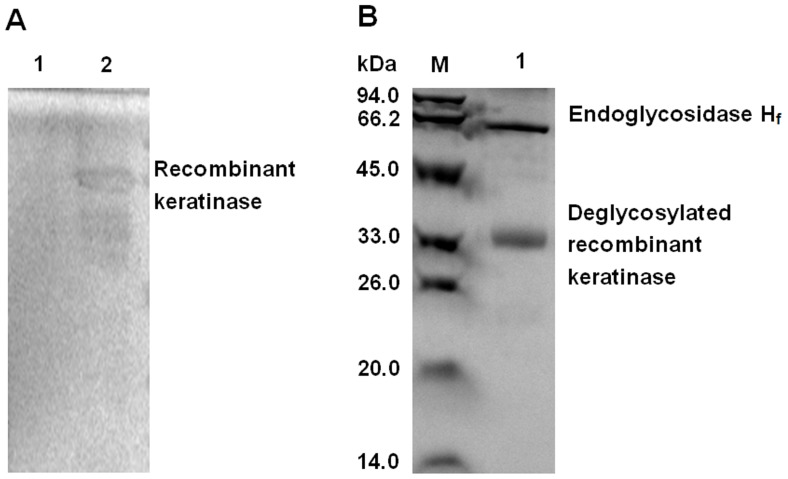
The glycoprotein staining and deglycosylation of purified recombinant keratinase in the SDS-PAGE. A: The glycoprotein staining experiment. Lane 1: BSA; Lane2: purified glycosylated keratinase. B: The deglycosylation of purified recombinant keratinase. Lane M: protein MW markers (14–94 kDa); Lane 1: deglycosylated keratinase.

Further, the SDS-PAGE analysis with glycoprotein staining and deglycosylation experiment was used to determine the possible glycosylation of the recombinant keratinase in *P. pastoris* system attributing to the increased molecular mass. Figure 4A (lanes 2) showed there was a clear band on SDS-PAGE by glycoprotein staining. After deglycosylation of the recombinant keratinase with Endoglycosidase H_f_, it presented a single band of approx. 33 kDa in size (Figure 4B lanes 1) which was similar to previous studies [8].

### Recombinant Keratinases Production by *P. pastoris* X-33

**Figure 5:**
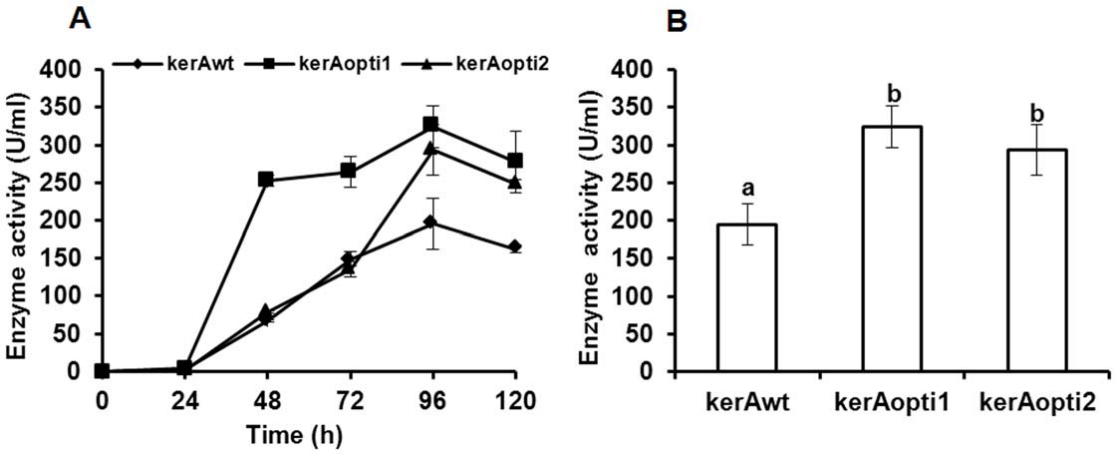
Recombinant keratinases production by *P. pastoris* X-33. A: Kinetics of keratinase production by *P. pastoris* pPICZα-kerAwt, pPICZα-kerAopti1 and pPICZα-kerAopti2. B: Comparative expression of *kerAwt*, *kerAopti1* and *kerAopti2* in *P. pastoris* at 96 h. The values represent means ± standard deviations (SD) of six independent positive transformants. Every transformant was examined in four independent experiments in quadruplicate. ^a,b^Mean values were significantly different (P<0.05) when labeled with unlike letters.

**Figure 6:**
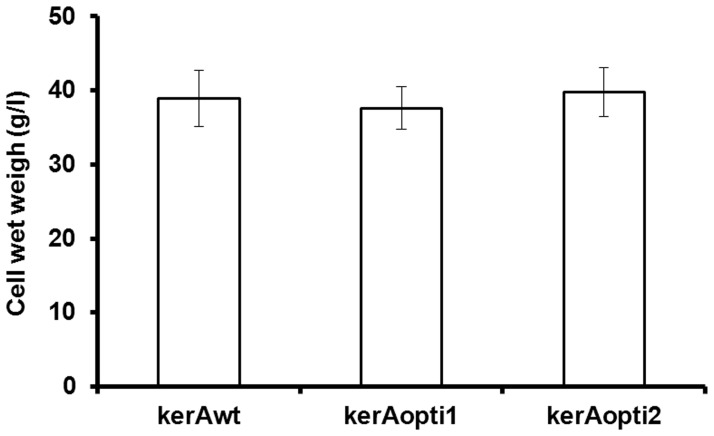
Comparative biomass concentration of *P. pastori* pPICZα-kerAwt, pPICZα-kerAopti1 and pPICZα-kerAopti2 at 96 h. The values represent means ± SD (n = 3). Results showed there was no significant difference in biomass concentration of every positive transformant (P>0.05).

The positive *P. pastoris* transformants were cultivated in BMMY broth at 29°C and induced by methanol. Keratinolytic activity was detected after 24 h (Figure 5A). The highest keratinase activity produced by *P. pastoris* pPICZαA-kerAwt, pPICZαA-kerAopti1 and pPICZαA-kerAopti2 was 195 U/ml, 324 U/ml and 293 U/ml respectively at 96 h (Figure 5B). In addition, figure 6 showed there was no significant difference in biomass concentration of all recombinant transformants (P>0.05).

The keratinase gene of *Bacillus licheniformis* S90 was optimized for the industrial application value in improving its production and for investigating the effect of different codon optimization strategies on the expression of *kerA* in *P. pastoris*. Many studies have suggested that codon biases reduce the metabolic load due to decreasing the diversity of isoacceptor tRNAs and consequently improve heterologous gene expression in host [17], [31]. In addition, the presence of two consecutive rare codons which ought to induce ribosomal frameshift during translation will inhibit the protein expression [17], [32]–[33]. Comparison of *P. pastoris* codon usage [34]–[36], there were four *P. pastoris* rare codons CCG (Pro-3), GCG (Ala-4), CGC (Arg-322) and CGT (Arg-324) which should decrease the enzyme expression level in native *kerA* gene. Schutter et al. (2009) have suggested that the synonymous codon usage bias technique was effective on increasing the expression of foreign proteins [35]. Yao et al. (1998) and Chen et al. (2005) have indicated that the production of phytase gene was great improved in *P. pastoris* by changing consecutive rare codons [37]–[38]. In this work, the results showed that the keratinase produced by *P. pastoris* pPICZαA-kerAopti1 (324 U/ml) and pPICZαA-kerAopti2 (293 U/ml) was markedly higher than the keratinase activity from recombinant *P. pastoris* pPICZαA-kerAwt (195 U/ml) (P<0.05, shown in Figure 5B). However, no significant difference in keratinolytic activity was found between *P. pastoris* pPICZαA-kerAopti1 and pPICZαA-kerAopti2 (P>0.05, shown in Figure 5B). The reason was probably that the expression level in heterologous expression systems was much lower if the rare codons locate at the N-terminal part of the protein than in other positions [17]. To our knowledge, the activity of keratinase produced from *P. pastoris* pPICZαA-kerAopti1 is significantly higher than most other reported results (Table 3).

**Table 3:** Comparison of keratinase activity from different source.

Enzyme source	Activity(different substrate, U/ml)		Reference
	α-keratin (keratin azure)	β-keratin (feather meal or azokeratin)	
*B. licheniformis* MKU3	55.0	NA^a^	[12]
*E. coli* pETproK3	74.3	NA^a^	[12]
*B. megaterium* pWHK3	95.0	NA^a^	[12]
*P. pastoris* pPICZαA-kerA	NA^a^	285.0	[13]
*P. pastoris* pPZK3	135.0	NA^a^	[9]
*P. pastoris* pPICZαA-kerAopti1	320.0	590.0	This study

a NA, not available.

### Biochemical Properties of the Purified Keratinase

**Figure 7:**
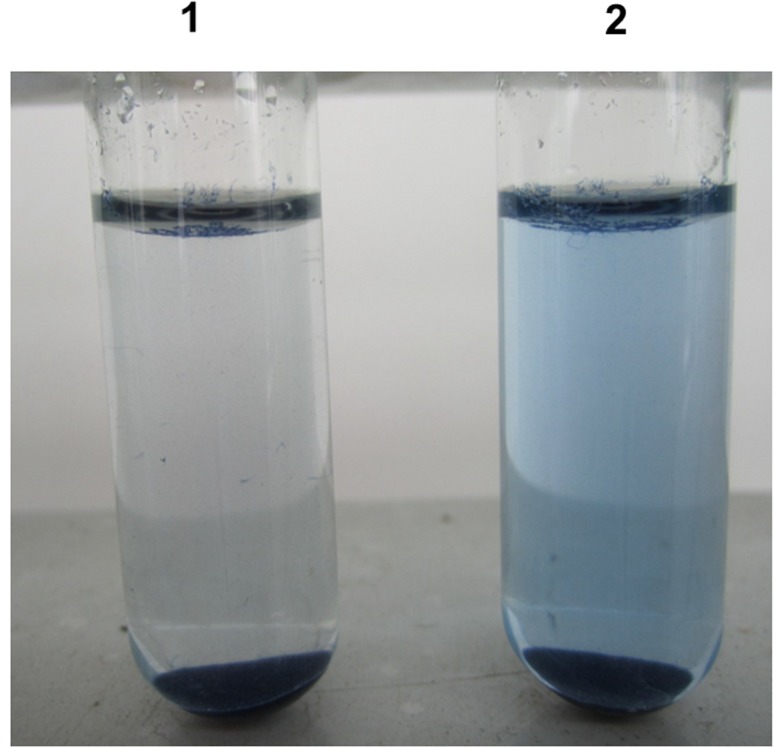
The keratin azure degradation by recombinant keratinase at 50°C after 1 h (1, control without keratinase; 2, keratinase treatment).

**Figure 8:**
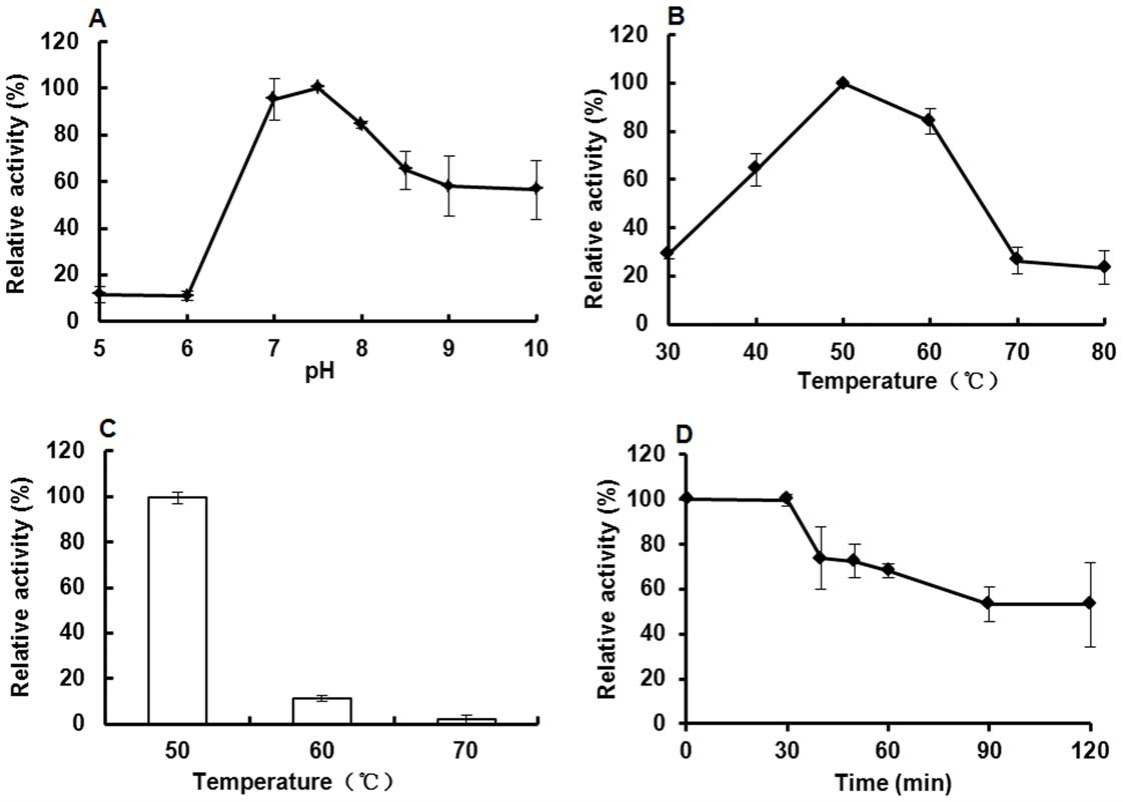
Enzymatic properties of the purified keratinase. A: The optimum pH of keratinase; B: The optimum temperature of keratinase; C: The keratinolytic temperature stability; D: The keratinolytic thermostability. The maximum value was taken as 100%.

The enzymatic characterization of the purified recombinant keratinase was detected using keratin azure as substrate (Figure 7). The recombinant enzyme was active at pH values from 7–9 and had optimum pH of 7.5 (Figure 8A), which is the same as the keratinase of *B. licheniformis* or other recombinant keratinases [8]–[9], [12]. In addition, the recombinant keratinase was stable at moderate temperature and had optimum temperature at 50°C (Figure 8B), which was also identical to the previous reports [4], [8]. The recombinant keratinase is much more stable at optimum temperature (50°C), remained approx.50% of the maximum activity at this temperature for 2 h. However, the keratinase from *P. pastoris* was rapidly inactivated at higher temperatures 60–70°C for 30 min (Figure 8C, D).

### Enzymatic Hydrolysis of Different Keratin

**Figure 9:**
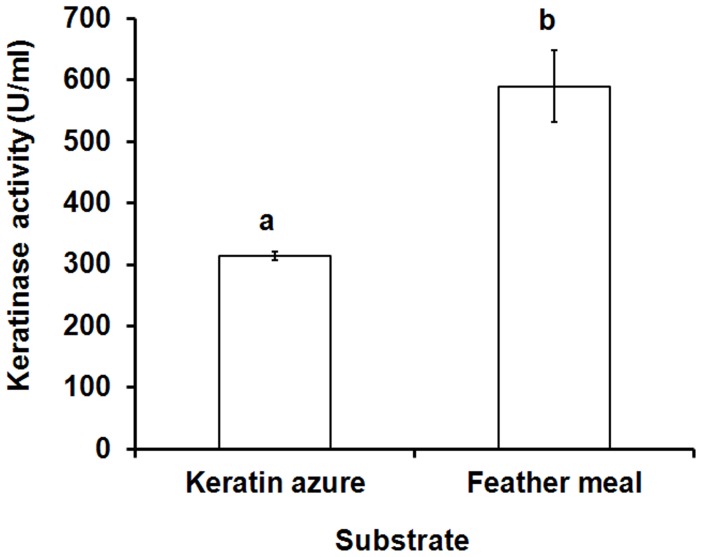
Enzymatic hydrolysis of different keratin. The values represent means ± standard. ^a,b^Mean values were significantly different (P<0.05) when labeled with unlike letters.

The hydrolytic activity of the recombinant keratinase on α-keratin (keratin azure) and β-keratin (chicken feather meal) was determined (Figure 9). When the substrate chicken feather meal was used, the yield of enzymatic hydrolysis was significantly higher than the substrate keratin azure. These results confirmed that the recombinant keratinase could effectively degrade both α-keratin and β-keratin [8], [10]. The reason was probably that the keratinase preferably cleave aromatic or hydrophobic amino acids at the P1 site [4], [10].

### Conclusions

The main *kerA* from *B. licheniformis* S90 was designed by two codon optimization strategies and successfully expressed in *Pichia pastoris*. The recombinant *P. pastoris* pPICZαA-kerAopti1 secreted more keratinase (324 U/ml) than *P. pastoris* pPICZαA-kerAwt, pPICZαA-kerAopti1 and most other reported results. The broad pH profile, substrate specificity and thermal stability make the recombinant keratinase from *P. pastoris* pPICZαA-kerAopti1 a suitable applicant for various industrial applications.
